# Metal Knitting: A New Strategy for Cold Gas Spray Additive Manufacturing

**DOI:** 10.3390/ma15196785

**Published:** 2022-09-30

**Authors:** Rodolpho F. Vaz, Vicente Albaladejo-Fuentes, Javier Sanchez, Unai Ocaña, Ziortza G. Corral, Horacio Canales, Irene G. Cano

**Affiliations:** 1 Thermal Spray Centre CPT, Universitat de Barcelona, Carrer Martí i Franques 1, 7a planta, 08028 Barcelona, Spain; 2 Cátedras CONACyT—Centro de Ingeniería y Desarrollo Industrial (CIDESI), Av. Playa Pie de la Cuesta No. 702, Desarrollo San Pablo, Santiago de Querétaro C.P. 76125, Mexico

**Keywords:** cold gas spray, strategy, Metal Knitting, additive manufacturing, geometries

## Abstract

Cold Spray Additive Manufacturing (CSAM) is an emergent technique to produce parts by the additive method, and, like other technologies, it has pros and cons. Some advantages are using oxygen-sensitive materials to make parts, such as Ti alloys, with fast production due to the high deposition rate, and lower harmful residual stress levels. However, the limitation in the range of the parts’ geometries is a huge CSAM con. This work presents a new conceptual strategy for CSAM spraying. The controlled manipulation of the robot arm combined with the proper spraying parameters aims to optimize the deposition efficiency and the adhesion of particles on the part sidewalls, resulting in geometries from thin straight walls, less than 5 mm thick, up to large bulks. This new strategy, Metal Knitting, is presented regarding its fundamentals and by comparing the parts’ geometries produced by Metal Knitting with the traditional strategy. The Metal Knitting described here made parts with vertical sidewalls, in contrast to the 40 degrees of inclination obtained by the traditional strategy. Their mechanical properties, microstructures, hardness, and porosity are also compared for Cu, Ti, Ti6Al4V, 316L stainless steel, and Al.

## 1. Introduction

Manufacturing industry is always looking for new cost-effective processes showing minimal raw material consumption, zero waste generation, and minimum energy consumption. Therefore, it promotes the rise of sustainable and more efficient technologies capable of responding to these needs [1]. During recent decades, many alternatives to traditional production methods have arisen in the market with the capabilities to fabricate unique components made in special alloys, which are not feasible by the conventional fabrication processes due to production complexity, properties, or even the high costs [2]. Beginning with polymer 3D printing in the 1980s and advancing its concept to producing metallic parts, the Additive Manufacturing (AM) techniques have expanded greatly in this period. They have been presented as an alternative to satisfy some of the above-mentioned industry and societal needs. A simple research in Scopus’ database can demonstrate the relevance of these technologies, where the number of documents published per year using the query “additive AND manufactur*” has grown from 278 works in 2000 to 781 in 2010 and then 10,751 in 2021, an extraordinary increase of 1300% in only one decade.

In the case of metallic AM components, some processes have become widespread, such as arc welding, laser melting or sintering, and cold gas spray (CS), among other methods [3,4,5]. Each process has different characteristics, limitations, or pros and cons, e.g., laser processes can produce very complex geometries but slowly and generate tensile residual stress in the material [6]. The welding or assembling of small AM individual parts is proposed in the literature as the best option to produce a large component [7]. However, this welding may result in distortion, shrinkage, or high tensile residual stress in the final AM part. Nevertheless, most of these AM techniques are based on the melting of raw material, which is an issue for some materials, such as the oxygen-sensitive Ti alloys, limiting the application of the technology.

### 1.1. Cold Gas Spray as an Additive Manufacturing Process

CS is a method of depositing powdered materials in the solid state, in which the cohesion of powder particles in the deposit is generated because of their impact under supersonic velocity onto a substrate/surface. For this, the CS equipment heats a gas in a chamber, discharging it under high pressure through a De Laval nozzle and accelerating it to supersonic velocity, dragging the feedstock powder under its recrystallization temperature [8,9].

The literature presents the application of CS for AM for a large number of materials, highlighting the components made from 316L stainless steel [10,11,12,13,14], Cu alloys [15,16,17], and Ti alloys [18,19,20,21], but not limited to them since Al alloys [21,22,23,24], Ni superalloys [25,26], maraging steel [27,28,29], and many others have also been studied. Moreover, these CSAM components have interested different sectors, such as aerospace, automotive, energy, medical, and marine sectors, among others [8,30]. In this sense, Prashar and Vasudev [31] list the CSAM benefits for sustainable manufacturing: optimizing product structures and designs, no environmental effect, component restoration rather than replacement, and improving the valuable life service of the component. The scientific interest in CSAM can be confirmed by a simple search in Scopus’ database searching by the query “additive AND manufactur* AND cold AND spray*” OR “3d AND print* AND cold AND spray*”, resulting in zero documents until 2011, 12 in 2015, 86 in 2020, and 81 in 2021, increasing by 675% the number of documents in the last five years.

In recent years, most of these studies have focused on CS mechanical properties and high deposition efficiency (DE). DE is the ratio between the CS sprayed powder mass and the consolidated part mass, which is directly influenced by the velocity reached by the particles when impinging the surface. Regarding CS parameters, adjustments and variations in CS working gas temperature and pressure, the nozzle design and geometry, and the spraying stand-off distance have been effective for improving the DE particle cohesion. It is widely accepted that the sprayed particles have to impact the substrate with a velocity between a critical or minimum and a maximum limit value.

### 1.2. CSAM Deposition Strategies

**Figure 1:**
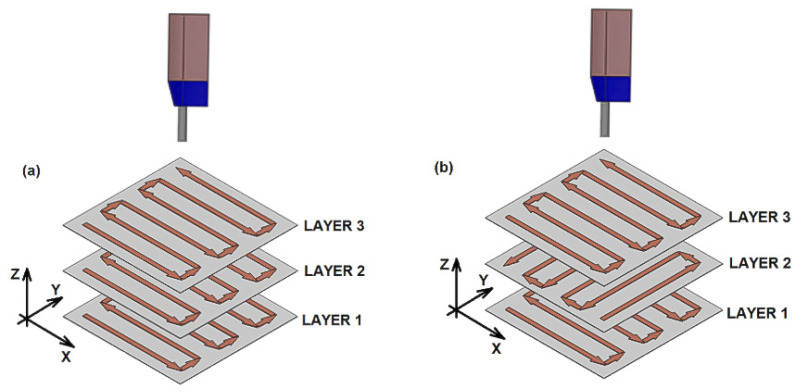
Strategies of robot path for CSAM, (**a**) traditional or bidirectional and (**b**) cross-hatching.

**Figure 2:**
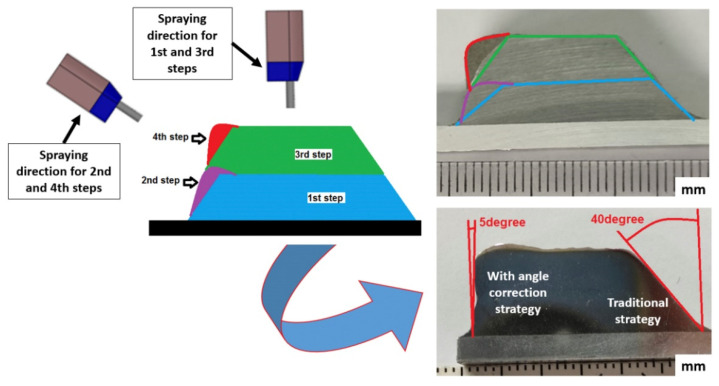
Scheme of strategy to compensate the sidewall inclination. The CSAM part cross-section is 316L on Al substrate.

The traditional strategy used in CS for powder deposition is based on a linear and normal movement of the CS gun with respect to the substrate surface, which might repeat for depositing consecutive layers, or alternating this direction, as presented by arrows in Figure 1. This extended deposition strategy promotes the growth of CSAM part sidewalls off-normal angle with the substrate surface, which limits the use of CSAM to near-net-shape components production that should finally be post-machined until the desired geometry and dimensions are obtained. The inclination shown by CSAM part sidewalls usually leads to more or less slope depending on the material sprayed, CS spraying parameters, or the substrate geometry, such as spraying on its flat surface or edges. The literature presents some approaches to developing a suitable deposition strategy for CSAM. For instance, some authors have proposed that it is possible to compensate this inclination in consecutive spraying tests, making the particles laden jet perpendicular to this inclined surface [32,33,34], Figure 2. Here, it is mandatory to know the inclination angle of the growth sidewall and redress the geometry at the end. However, this strategy is not feasible when thin walls are intended. In addition, the modification of the spraying directions generates new interfaces in which the cohesion of the particles is weaker, promoting an inhomogeneity of final component properties.

The use of CSAM has grown in recent years. Some researchers have developed strategies to control the CSAM parts geometries, mainly regarding sidewall inclinations because this is a considerable limitation for CSAM in producing freeform parts. The presence of undesired sidewall inclination represents a loss of spraying time and raw material, besides needing more post-machining time. An efficient alternative seen in the literature is the correction of the sidewall inclination by a sequence of layers with different CS jetting angles, Figure 2. However, it demands a specific robot strategy or parameters for each material and CS spraying condition. Thus, in this work, the authors aim to present the effectiveness of using a new CSAM deposition strategy in substitution for the CSAM traditional strategy commonly used. The strategies are compared not only by the CSAM geometries produced, mainly the sidewall angles inclination, but they are also evaluated regarding the microstructures obtained, and their physical and mechanical properties. The novelty in the Metal Knitting strategy is developing a robot strategy capable of producing thin vertical walls or large bulks, thereby eliminating geometry correction steps, which are demanded when using the traditional strategy.

## 2. Materials and Methods

316L stainless steel, pure Cu, pure Al, Ti grade 2 (pure Ti), and Ti grade 5 (Ti6Al4V) powders were sprayed using the newly developed and traditional CS strategies. Irregular and spherical water atomized powder were used in these tests in order to show the capabilities for generating controlled geometries by Metal Knitting using powder with different morphologies. The feedstock powders’ particle size distribution was measured by laser scattering in LS13320 (Beckman Coulter, Brea, CA, USA), and the particles’ shapes were interpreted from SEM images obtained in equipment Pro Desktop (Phenon, Eindhoven, The Netherlands). The feedstock powders’ chemical composition was measured by Inductively Couple Plasma (ICP) in equipment Optima ICP-OES 3200 RL (Perkin Elmer, Waltham, MA, USA) and is presented in Table 1.

**Figure 3:**
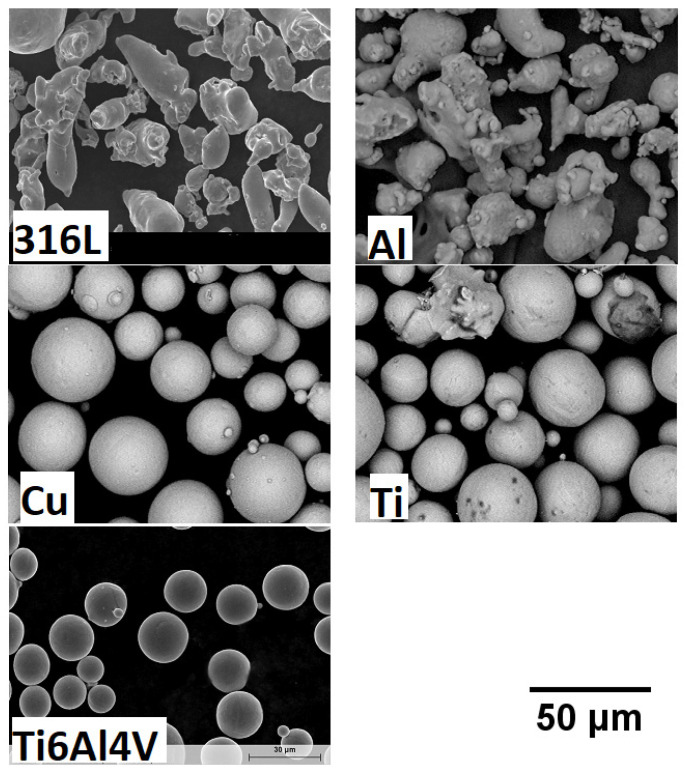
SEM images of feedstock powders for CSAM.

**Figure 4:**
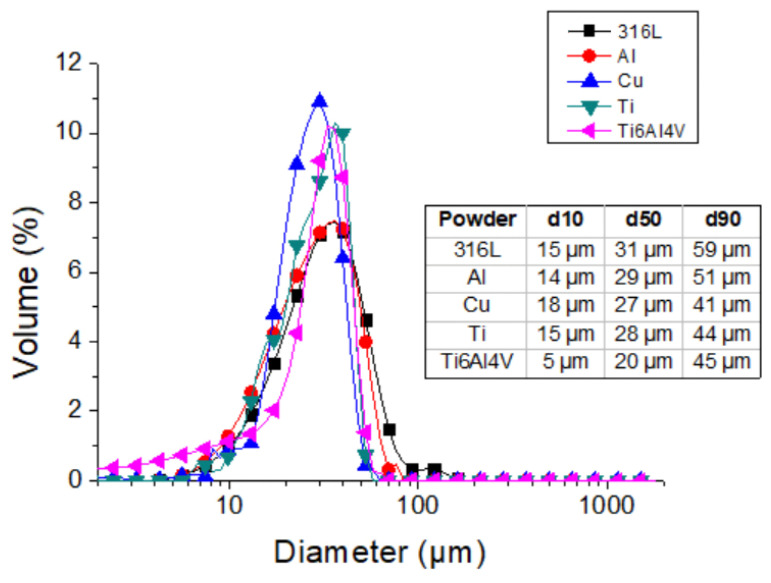
Feedstock particle size distribution for CSAM.

The five feedstock powders for CSAM have the shapes obtained by SEM presented in Figure 3, where it is possible to see the typical irregular shape for the water-atomized ones, 316L and Al, as well as the spherical gas-atomized Ti alloys and pure Cu. The powder size distribution is also presented in Figure 4, which is quite similar for all the powders and is in the range optimal for CS processes because particles out of this range may not reach the optimal velocity at the impact on the substrate [35,36].

Regarding the particle shape, the water-atomized 316L and Al are cheaper options than gas-atomization powders since the production costs are lower for this manufacturing process. The raw material price is an important factor when applying the CSAM technique due to its heavy impact on the part‘s final costs. Previously, Vaz et al. [10] presented for CS 316L coatings the quite similar performance of gas-atomized and water-atomized powders, regarding mechanical properties, wear resistance, and corrosion behavior. It supports the selection of the irregular powder used in this article.

The CSAM depositions were performed using PCS100 equipment(Plasma Giken, Saitama, Japan) and N_2_ as working gas. The main spraying parameters are indicated in Table 2. The robot was an IRB 2400 M2004 (ABB, Västerås, Sweden) and the programming was performed in ABB RobotStudio v6.08.01 software. The PCS100 gun was kept static while the sample holder performed all the robot arm movements. For the CSAM samples deposited by the traditional strategy, the robot path followed the Figure 1a scheme, with velocity of 500 mm·s^−1^.

The CSAM parts produced were evaluated regarding the geometries obtained, sidewall angles, and visual characteristics. In addition, a visual inspection was performed to find CSAM part imperfections, such as cracks, delamination between the layers, and adhesion to the substrate, among others. The microhardness of samples was measured by means of HMV equipment, applying a load of 0.3 kgf (HV0.3) and presenting a mean value of 10 values for each sample. The metallographic preparation was carried out in accordance with the ASTM E1920-03 and ASTM E3-01 standards, and the porosity was analyzed from light microscopy images obtained in Leica DMI3000 M microscope at 200× *g* magnification, according to ASTM E2109-01 standard. For tensile testing, three samples of each CSAM part were manufactured by the wire Electrical Discharge Machining (EDM) process. A Zmart.Pro equipment(ZwickRoell, Eisengen, Germany) with an Xforce P 10 kN load cell was used for the tensile testing, with a load application velocity of 1.0 mm·min^−1^.

## 3. Results and Discussions

### 3.1. Influence of Substrate Shape on CSAM Part Geometry

**Figure 5:**
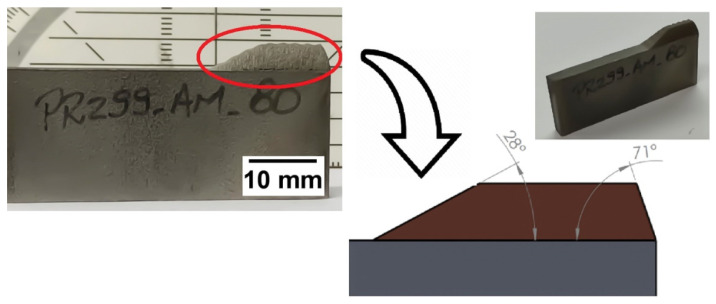
CSAM 316L growing from a substrate’s flat surface or from its edge.

Before evaluating the effect of the new strategy for geometric control of CSAM deposits, 316L deposits using an irregular powder were produced by the traditional strategy that will be considered a CSAM benchmark for the Metal Knitting samples evaluations. Figure 5 shows a CSAM 316L deposit sprayed onto a carbon steel plate’s thinnest face (5 mm) to highlight the effect of the traditional spraying strategy on the inclination of the sidewalls generated. Here, it can be observed that the CSAM sidewalls inclinations are different for each substrate area or region scanned, considering the substrate’s edge or flat surface. For example, from the substrate’s edge, the CSAM 316L part grew up at 71 degrees relative to the substrate’s flat face, while it grew at an angle of 28 degrees from a flat surface. Table 3 shows the sidewall angles obtained for 316L, Al, Cu, Ti, and Ti6Al4V as well. These results indicate that this inclination effect is dependent on the powder material sprayed, and not limited to stainless steel or irregular feedstock materials.

**Figure 6:**
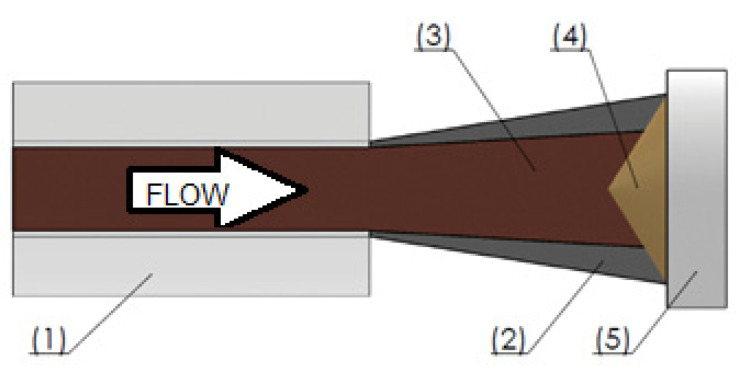
Scheme of CS particles laden jet. (1) Nozzle, (2) sparse particles and lower velocity particles laden jet, (3) concentrated particles and high-velocity particles laden jet, (4) sprayed material, (5) flat substrate.

It is widely accepted that the velocity and the density of particles in the center of CS particles laden jet flow are higher than in its periphery [9,24,37,38,39]. A lower velocity and number of particles in the periphery of the CS particles laden jet produce a progressive thinner layer at the border of the sprayed layer (Figure 6), promoting a thickness distribution or single-track profile defined by a Gaussian distribution [40]. This effect explains the inclined sidewalls commonly obtained by the traditional spraying strategy in CSAM. The behaviour observed in different regions of the substrate is explained by considering the geometric features of the sample [38,41] since, for a deposition in a substrate edge, the CS particle plume passes completely through this edge, finishing its travel out of the sample, where the robot changes the direction and turns back to another pass. However, this does not occur for the deposition on a flat surface, where particle distribution in the CS particle plume always affects the formation and consolidation of CSAM part geometry because all the changes in robot movement directions are done on the substrate surface.

Besides considering the density of particles in different positions of the CS plume, it is worth considering that the farther the particles are radially from the jet center, the lower their velocities are, Figure 6. It is well known that the CS deposition and the part consolidation depend strongly on the velocity of the particles [42,43,44], but radially just a few millimetres from the CS jet center, the particles present a severe reduction in velocity, even to out of the CS deposition window of velocity. Previous CFD simulations by several authors have predicted this behavior under specific CS parameters. For example, Al and Ti particles have a velocity of 700 m·s^−1^ close to the particles laden jet center, but radially 4 mm far from this center, the velocity falls to 500 m·s^−1^, a reduction of almost 30% [45,46,47]. For 316L, this trend is the same [48], which influences the CSAM part consolidated geometry.

Consequently, a not constant particles-laden jet is deposited onto the sprayed surface from the very beginning of the CS process, at the very first layer, which leads to the generation or growth of inclined sidewalls or geometries. This result can be directly correlated with a lower DE of particles in the periphery of the CS particles laden jet because of their above described lower impact velocity, producing a progressive thinner layer at the border of the sprayed layer [32]. Then, when subsequent particles reach the substrate, the previous sprayed material, with different angles at the edges, modifies the normal impact angle of the particles with the surface, which leads to a reduction of the DE and promotes the sidewall inclination layer by layer [32,38,39]. Higher impact angles reduce the velocity component perpendicular to the substrate by the sine of this angle, which is responsible for the particles’ adherence reduction [38]. Rokni et al. [49] presented an insignificant change in particle properties by CS jet axis up to 20 degrees off-normal angle with the substrate. Li et al. [38] showed that a 30 degrees angle was enough to reduce DE from close to 100% to 40% for Al, and 50% for Cu, as an angle of 70 degrees promoted a DE of zero. The other velocity vector component, related to the cosine of the impacting angle, is a tangential velocity, which collaborates with the inclination of the CSAM part sidewall by an erosion mechanism [34,50].

Thus, in traditional CS spraying strategies based on a normal impact of particles onto the substrate or surface, an inclined CSAM part surface is established, due to the difference in velocity and density of particles in the CS particles laden jet periphery compared to its canter. After a few layers of material deposition, this effect promotes the CS sprayed particles reaching the new target at off-normal impact angles, progressively reducing the DE in the periphery of the particles laden jet. Table 3 summarizes the sidewall angles produced by CSAM using traditional and Metal Knitting strategies. The higher control in this geometric CSAM part characteristic evidently results from the use of Metal Knitting, highlighting the depositions on a flat surface; e.g., Cu had 75 degrees with the vertical line by using traditional strategy versus zero degrees by employing Metal Knitting. However, this discrepancy of values was not seen on edges due to the reasons above-mentioned.

### 3.2. The Metal Knitting Strategy

**Figure 7:**
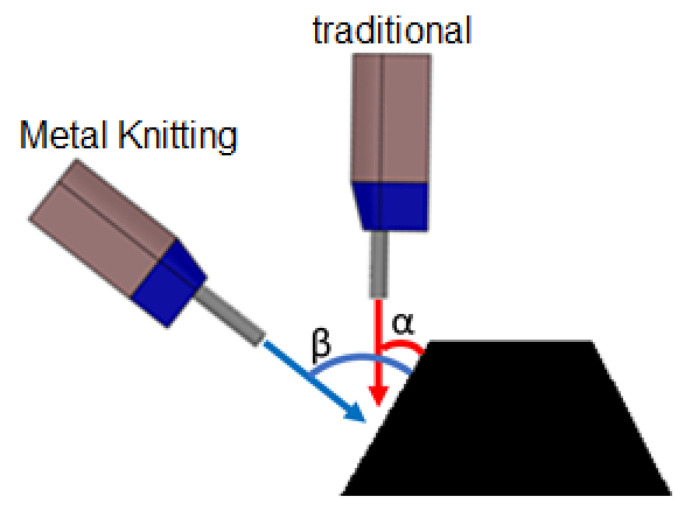
Scheme of the angle of impact of particles on an inclined surface by traditional (α) and Metal Knitting (β) strategies.

The brand-new strategy presented in this work is named Metal Knitting, relating to the movements of needles dealing with yarn to make a woven fabric, which drastically modifies the relative gun/substrate movement from the traditional movements presented in the literature for the CSAM process to date. This new strategy aims to make the CS sprayed particles reach the substrate or the growing CSAM part with an angle that maximizes the DE at the sidewalls, simultaneously controlling the geometry growth of the CSAM part, Figure 7.

**Figure 8:**
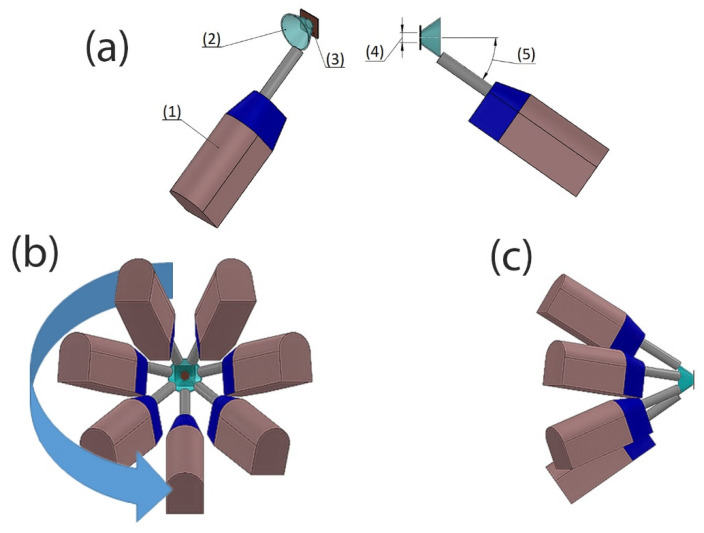
Metal Knitting strategy scheme. (**a**) (1) CS gun, (2) virtual frustum of cone path, (3) substrate, (4) radius, (5) angle. (**b**,**c**) simulation of different directions of view of CS gun describing the Metal Knitting movements.

For this purpose, the Metal Knitting strategy impresses a circular-like movement on the substrate plane. Still, the CS particles laden jet is not perpendicular to this plane. The final path is a virtual frustum of a cone, with the axis of rotation perpendicular to the substrate plane, as presented schematically in Figure 8. To make clear the Metal Knitting robot movements, the Video S1 is available as a supplementary material to this work.

#### 3.2.1. Metal Knitting Parameters

The variables or parameters for Metal Knitting strategy and their influences on the CSAM part characteristics include:

- Velocity of the robot: the linear or tangential velocity described by the gun in the circular-like trajectory. The higher the velocity, the thinner the CSAM layer;
- Radius: the distance from the center of the circle to the circumference described by the particles laden jet center on the substrate surface plane, or the small radius of the virtual frustum of the cone path. Excessive radius value generates unevenness in the layer shape;
- Step: the distance from a circle to an adjacent circle. Excessive step value results in waves-like topography of the layer;
- Angle: the angle between the axis of rotation and the frustum of the cone generator described by the gun movement. Excessive angle reduces the DE by the powder’s velocity component reaching the substrate.

#### 3.2.2. The Metal Knitting Deposit Properties

**Figure 9:**
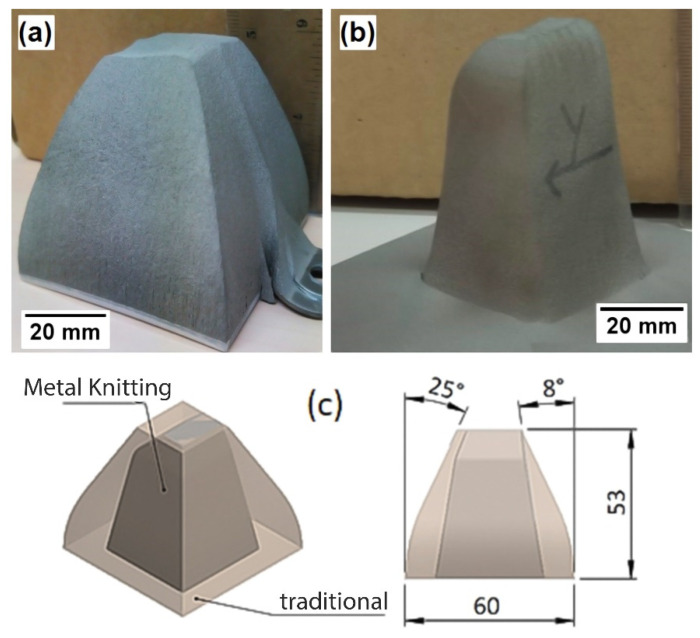
CSAM 316L bulks. (**a**) traditional strategy on the substrate edges and (**b**) Metal Knitting strategy on flat substrate. (**c**) scheme comparing the geometries obtained.

A comparison between the CSAM 316L bulks produced by traditional and Metal Knitting strategies is presented in Figure 9, which also shows the real bulk part obtained. Identical deposits were CSAM fabricated with Ti and Ti6Al4V alloy powders with successful results. Visual inspection showed that none of the bulk parts produced (316L, Ti, and Ti6Al4V) presented any cracks or detachments from the substrate. In addition, the parts made by Metal Knitting showed smoother surfaces and more rounded edges compared to the rough finishing and sharp edges seen in the CSAM part produced by the traditional spraying strategy. In this sense, using the Metal Knitting strategy on the substrate’s flat surface, CSAM 316L, Ti, and Ti6Al4V components showed a sidewall angle of around 80 degrees to the substrate surface. It is a significantly smaller inclination value than the inclination produced by the traditional strategy on a flat surface, as shown in Figure 9.

As indicated above, in CS deposition by the traditional strategy, the angle of impact of subsequent particles is always off-normal from the beginning of the process. In contrast, the Metal Knitting strategy can keep this particle impact angle constant throughout the process. As a result, during the cone-like Metal Knitting movement, the CS particles laden jet reaches the substrate with angles that promote higher DE in the side walls. It occurs by improving the velocity vector component normal to the impacting surface, as seen in the scheme presented in Figure 7, which improves the capabilities of the CSAM process to control the final geometry generated.

**Figure 10:**
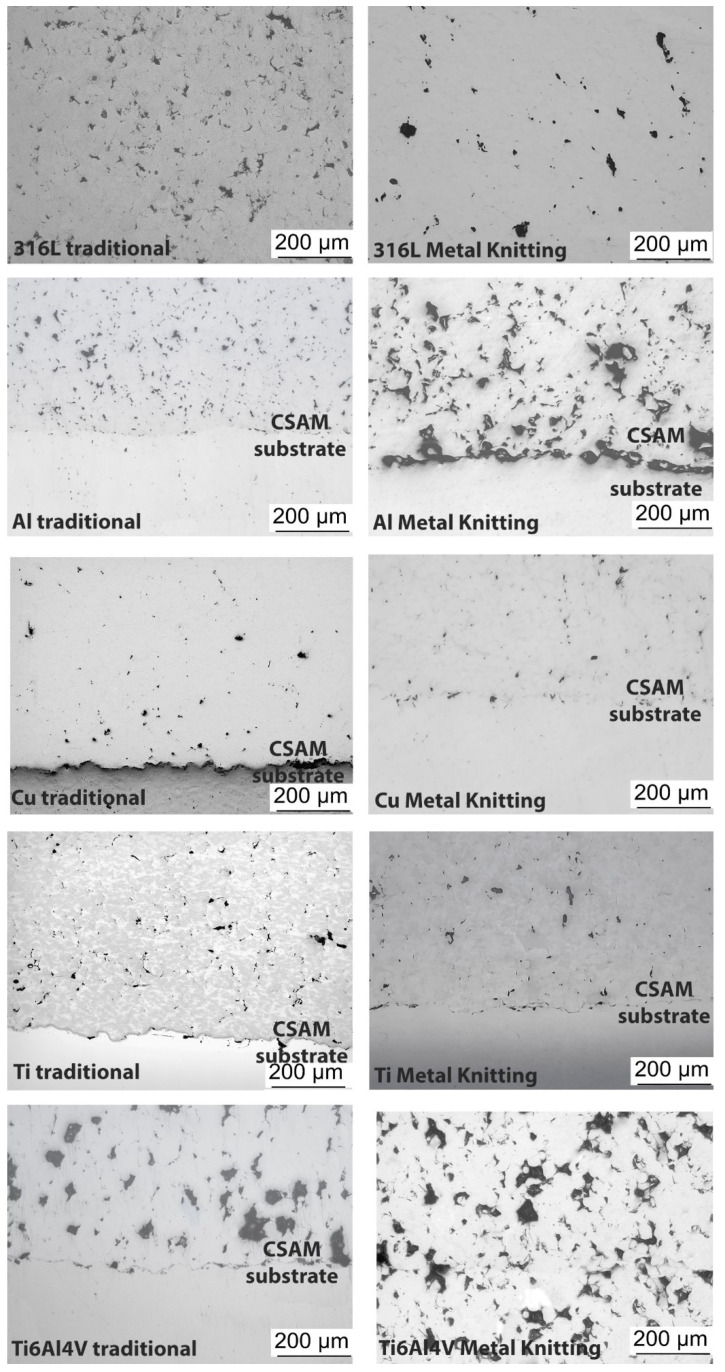
CSAM microstructures.

CSAM parts or any material obtained by CS have properties influenced by the cold-working, resulting from the severe plastic deformation under the material’s recrystallization temperature. This increases the density of dislocations in the metal’s crystalline structure and consequently improves the properties of the sprayed feedstock material. Figure 10 presents the as-sprayed CSAM microstructures obtained by Metal Knitting and traditional strategies, which is in consonance with observations in the literature. There is a trend of higher density for the most ductile sprayed materials, Al and Cu, while harder materials resulted in a higher porosity, as seen for Ti6Al4V. This has influences on the material’s properties, as summarized in Table 4. For the CSAM samples made by the traditional strategy, the microstructures, hardness, and porosity obtained were close to those presented in the literature for 316L [10,13,14,51], Al [21,23,24], Cu [16,17,21,52,53], and Ti alloys [19,20,21]; however, for Metal Knitting the literature did not present any result, because it is a brand-new strategy.

The Metal Knitting materials’ properties must be evaluated by their comparison with the CSAM samples obtained by the traditional strategy. The CSAM Metal Knitting samples showed a higher porosity, but close hardness values. The higher discrepancy in hardness values was seen for the materials more susceptible to cold-working strain hardening, highlighting Ti and 316L, suggesting a higher deformation of particles for CSAM traditional strategy sprayed samples than for the Metal Knitting ones. On the other hand, Ti6Al4V had very close hardness values for both evaluated strategies, as expected by their similarity in microstructures.

Table 5 summarizes the tensile testing results of Ti6Al4V and 316L CSAM parts produced by Metal Knitting and traditional strategies. The latter had higher strength values because the cohesion of particles was higher, which is correlated with the lower porosity presented in Table 4. After all, the lower porosity infers a higher contact area among the particles of the CSAM consolidated material. Furthermore, the higher hardness also indicates a higher cold working by more severe particle deformation, which affects the material strength.

#### 3.2.3. CSAM Geometries Produced by Metal Knitting

**Figure 11:**
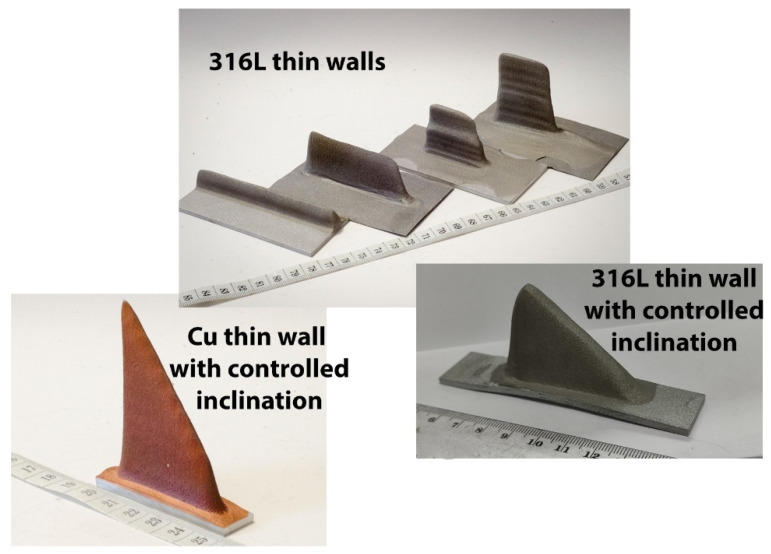
CSAM straight thin wall parts made by Metal Knitting strategy on flat substrate.

In order to show some capacities of this new spraying strategy, complex geometries were produced by CSAM Metal Knitting, which are not feasible by employing the CSAM traditional strategy. Thus, thin straight walls were obtained using the Metal Knitting strategy following one single-line on the substrate’s flat surface, as seen in Figure 11. The geometries presented are not practicable using the traditional strategy because the sidewalls grow with a certain relative inclination, resulting in a pyramid-like shape, while the CSAM sidewalls produced by a single-line Metal Knitting were perpendicular to the substrate surface, as indicated in Table 3.

Adjustments in the Metal Knitting parameters, radius, angle, and standoff distance, promote a variation in CSAM wall thicknesses. This change in the part wall thickness may be made during the part growing, producing an even more complex geometry with steps, like the two right-side 316L samples indicated in Figure 11 (316L thin walls).

Another approach for CSAM Metal Knitting was the variation in the number of steps by layer. The progressive reduction in this quantity of steps made possible the fabrication of a controlled inclined top wall, which is indicated for CSAM Cu and 316L in Figure 11 (Cu with controlled inclination) and Figure 11 (316L with controlled inclination), respectively, resulting in shark dorsal thin like geometries.

**Figure 12:**
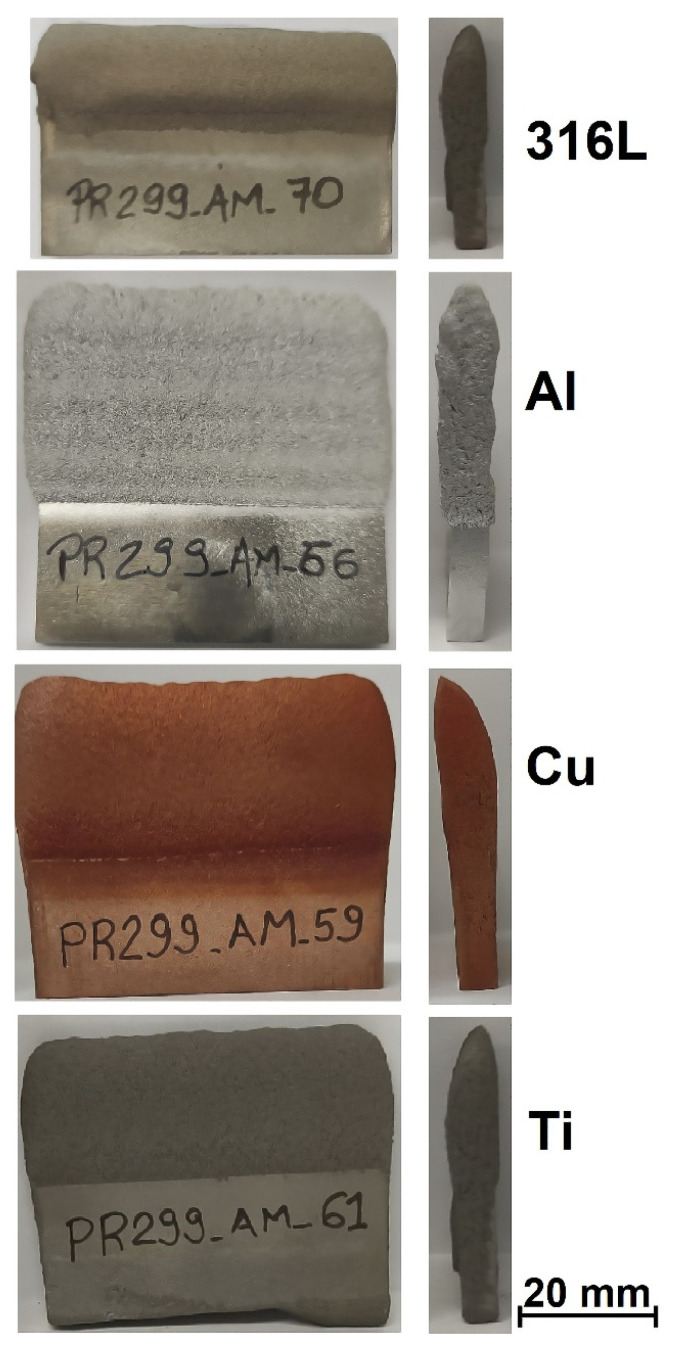
CSAM part on narrow substrate surface made by Metal Knitting strategy.

Additionally, the Metal Knitting strategy was used on a 50 × 5 mm substrate, a small area for CSAM, and the objective was to grow up the CSAM part with the same 5 mm wall thickness. For this deposition, the substrate and feedstock powder had the same composition, Cu, Ti, Al, and 316L, and a single-line Metal Knitting strategy was selected. The results showed how feasible it was to grow up a part with this 5 mm thick wall by the Metal Knitting strategy, as presented in Figure 12. Varying the Metal Knitting parameters, the 5 mm thick walls produced should be increased to 6 or 7 mm thick to produce sufficient material for a post-machining service.

**Figure 13:**
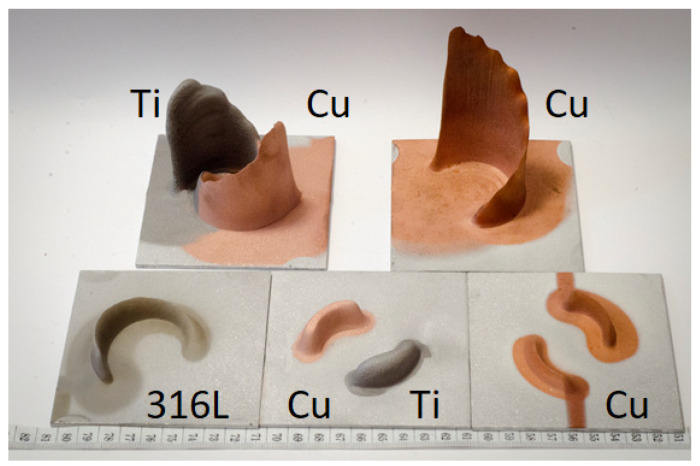
CSAM curved parts made by the Metal Knitting strategy.

The Metal Knitting was changed from a straight line based path to a curved lines based path, keeping the cone-like movements; following these non-straight lines, the production of curved walls was feasible, as presented in Figure 13. Different materials were tested, 316L, Ti, and Cu. A change in the robot program made it possible to grow multi-material close walls, Ti and Cu, on the same Al plate substrate.

The same approach presented in Figure 11 for promoting a controlled inclination for straight-line Metal Knitting 316L and Cu is indicated in Figure 13 for a curved path, growing the CSAM part with a controlled inclination. For CSAM 316L, it produced a height of 0 at the beginning of the wall and 16 mm at its end. However, reducing the number of steps per layer, a stairways-like wall was made, as seen in the highest Cu CSAM part in Figure 13. A control in the length of each pass promoted a smooth inclination of the wall, as seen for the 316L curved wall, with height 0 in the beginning of the wall, and 16 mm at its end. However, by decreasing more the length of each pass, a stairways-like top of the wall was made, as seen in the highest Cu CSAM part in Figure 13.

**Figure 14:**
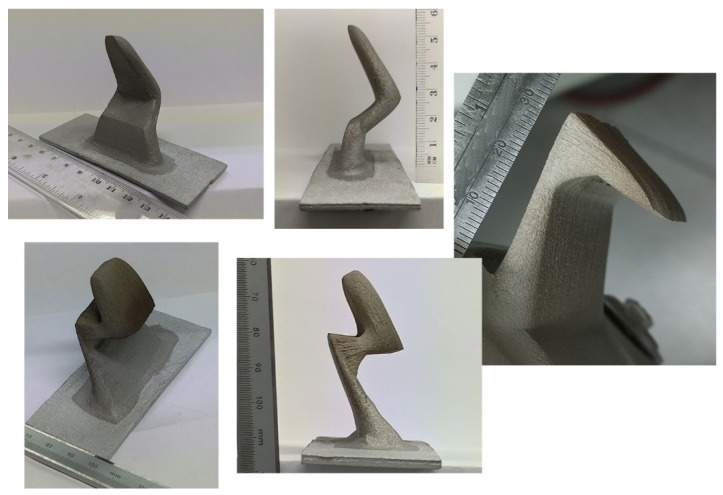
CSAM 316L complex geometries made by the Metal Knitting strategy.

The CSAM Metal Knitting strategy was also evaluated for more complex geometries, which were feasible by controlling the growth direction. Figure 14 presents CSAM 316L parts. For the left and right images of Figure 14, the spraying started in a direction non-perpendicular to the substrate, indicating a good adhesion because no cracks or delamination were observed after visual inspection. The changes in growing angle and direction were performed by the inclination of the substrate holder, making this activity easier because a change in the robot program for each of these geometries would demand much time and effort to make a unique part. However, a robot program can guarantee the dimensional quality requirements for mass production, or at least dozens of components.

## 4. Conclusions of Using the Metal Knitting CSAM Strategy and Future Perspectives

Comparing the Metal Knitting CSAM strategy to the traditional one, some points may be listed, mainly regarding future advances and possibilities of its use:

- Metal Knitting demonstrates the suitability for making geometries and dimensional capacities previously unfeasible by the traditional strategy, obtaining vertical sidewalls, despite 40 degrees of inclination in the 316L CSAM part produced by the traditional strategy;
- Metal Knitting produces materials with good mechanical properties and similar microstructures to the traditional CSAM strategy. In general, due to the constant cone-like CS gun movement, Metal Knitting results in a lower density than the traditional strategy;
- Post-processing for densification of Metal Knitting made parts have to be studied, e.g., heat treatments, Hot Isostatic Pressure (HIP), Spark Sintering Plasma (SPS), due to their effectiveness used for parts produced by the traditional CSAM strategy. This qualifies the parts made by Metal Knitting for quality improvement.
- Metal Knitting strategy is not necessarily a substitute for the traditional one. Still, it can be a complementary stage to this one, redressing the sidewalls to lower angles. Therefore, a combination of robot strategies could be a solution for specific applications, e.g., to build a large bulk, the traditional strategy can be applied for its central area, while knitting is used on the edges. This would prevent the sidewalls inclination and promote the best spraying angle deposition on the central location, which is 90 degrees, typical of the traditional strategy.
- As a CSAM technique, the Metal Knitting strategy has some challenges, e.g., needing reasonable control of its parameters, distances, and relative’ positions between the substrate and the CS gun, because a misunderstanding in the measurements and positioning may result in an undesired path and consequently unexpected geometry. Moreover, depending on the knitting path and part design, the robot arm does long movements, which might eventually result in a collision between the CS gun and the robot arm. Therefore, to avoid what can be catastrophic for the CS equipment, the robot user must check carefully and slowly the robot trajectory before the CS spraying.
- Looking for the potential applications of the CSAM metal knitting strategy, one possibility is using it to complement other AM techniques already in use, such as arc-welding or laser processes.
- In addition, Metal Knitting can be used for repairing elements, such as thin, worn, or damaged walls. Among a wide range of applications and industry sectors, some examples are: repairing Ti alloys turbine compressor blades’ tips, fabrication of superalloys manifolds, production of special prototypes for racing cars, fabrication and repairing of special alloys piping components for the oil and gas industry, repairing of eroded martensitic stainless-steel Kaplan hydraulic runners’ tips, and others.

## Figures and Tables

**Table 1:** Feedstock powders chemical composition (wt.%).

Powder	Cr	Ni	Mo	Mn	Si	Fe	Cu	Ti	Al	V
316L	16	12	2.5	0.5	<0.1	Bal.				
Cu							100			
Ti								100		
Ti6Al4V								Bal.	5.9	3.9
Al									100	

**Table 2:** CS spraying parameters.

Powder	N_2_ Temperature [°C]	N_2_ Pressure [MPa]	Standoff Distance [mm]
316L	1000	6.0	25
Cu	700	3.0	25
Ti	900	6.4	25
Ti6Al4V	1000	6.5	25
Al	450	3.0	25

**Table 3:** Sidewall angles produced by traditional and Metal Knitting strategies.

Material	Sidewall Angle Relative to a Line Normal to the Substrate [Degree]			
	on Substrate’s Flat Surface		on Substrate’s Edge	
	Metal Knitting Strategy	Traditional Strategy	Metal Knitting Strategy	Traditional Strategy
316L	8	60	0	20
Al	0	45	0	5
Cu	0	75	0	25
Ti	10	45	0	0
Ti6Al4V	10	40	0	5

**Table 4:** CSAM material properties produced by traditional and Metal Knitting strategies.

Material	Strategy	Hardness [HV0.3]	Porosity [%]
316L	Metal Knitting	246 ± 33	8
	Traditional	374 ± 26	4
Al	Metal Knitting	50 ± 3	21
	Traditional	49 ± 8	3
Cu	Metal Knitting	88 ± 10	7
	Traditional	109 ± 12	3
Ti	Metal Knitting	199 ± 22	10
	Traditional	262 ± 43	7
Ti6Al4V	Metal Knitting	229 ± 31	28
	Traditional	236 ± 32	14

**Table 5:** CSAM produced by traditional and Metal Knitting UTS results.

Material	Strategy	UTS [MPa]
316L	Metal Knitting	61 ± 2
	Traditional	184 ± 9
Ti6Al4V	Metal Knitting	32 ± 12
	Traditional	121 ± 25

## Data Availability

The data presented in this study are available on request from the corresponding author.

## Supplementary Materials

The following supporting information can be downloaded at: https://www.mdpi.com/article/10.3390/ma15196785/s1, Video S1: CSAM Metal Knitting strategy on flat substrate surface.
